# ﻿Two new oonopid spider species from Yunnan, China (Araneae, Oonopidae)

**DOI:** 10.3897/zookeys.1181.109597

**Published:** 2023-10-04

**Authors:** Shuhui Li, Dongju Bian, Yanfeng Tong, Zhisheng Zhang

**Affiliations:** 1 Life Science College, Shenyang Normal University, Shenyang 110034, Liaoning, China Shenyang Normal University Shenyang China; 2 Key Laboratory of Forest Ecology and Management, Institute of Applied Ecology, Chinese Academy of Sciences, Shenyang 110016, China Institute of Applied Ecology, Chinese Academy of Sciences Shenyang China; 3 Key Laboratory of Eco-environments in Three Gorges Reservoir Region (Ministry of Education), School of Life Sciences, Southwest University, Chongqing 400715, China Southwest University Chongqing China

**Keywords:** Goblin spiders, identification key, morphology, new record, taxonomy

## Abstract

The genera *Kachinia* Tong & Li, 2018 and *Promolotra* Tong & Li, 2020 are recorded from China for the first time. Two new species, *Kachinialongling***sp. nov.** (♂♀) and *Promolotralushui***sp. nov.** (♂♀) are described. Descriptions, diagnoses, photographs and keys to *Kachinia* and *Promolotra* species are provided.

## ﻿Introduction

The family Oonopidae Simon, 1890 includes 1893 extant species in 115 genera worldwide, mainly distributed in tropical regions (WSC 2023). Oonopid spiders are tiny, with a body size between 1.0 and 3.0 mm. They mainly inhabit leaf litter and similar habitats, such as woodrat nests, under rocks, or interstitially (Ubick and Dupérré 2017).

Currently, 125 oonopid species have been recorded in China, of which 37 species belonging to seven genera are known so far in Yunnan, China (Tong and Li 2015a, b; Liu et al. 2019; Sun et al. 2019; Tong et al. 2019, 2021; Huang et al. 2021; WSC 2023). While studying new material we found two new species belonging to two genera that were not known previously from China; the two new species are described below.

## ﻿Material and methods

The specimens were examined using a Leica M205C stereomicroscope. Details were studied under an Olympus BX51 compound microscope. Photos were made with a Canon EOS 750D zoom digital camera (18 megapixels) mounted on an Olympus BX51 compound microscope. Endogyne were cleared in lactic acid. Scanning electron microscope images (SEM) were taken under high vacuum with a Hitachi S-4800 after critical-point drying and gold-palladium coating. All measurements were taken using an Olympus BX51 compound microscope and are in millimeters. The terminology used in the text and figures follows Tong et al. (2018) and Tong and Li (2020). The type material is deposited in Shenyang Normal University (**SYNU**) in Shenyang, China (curator: Yanfeng Tong).

## ﻿Taxonomy

### ﻿Family Oonopidae Simon, 1890

#### ﻿Genus Kachinia

Taxon classificationAnimaliaAraneaeOonopidae

Tong & Li, 2018

D5D6C7C9-8FE7-58E6-B71E-7EB047775E02

##### Type species.

*Kachiniaputao* Tong & Li, 2018 from Myanmar.

##### Comment.

The genus belongs to the subfamily Oonopinae Simon, 1890. According to Tong et al. (2018), the genus is similar to *Brignolia* Dumitrescu & Georgescu, 1983 in the sclerotized and darkened palps of males and the shapes of T-shaped anterior sclerite and posterior receptacle.

##### Composition.

*Kachinialongling* sp. nov., *K.mahmolae* Tong & Li, 2018, *K.putao* Tong & Li, 2018

##### Distribution.

China (Yunnan), Myanmar.

### ﻿Key to *Kachinia* species

**Table:** 

1 (0)	Males	**2**
—	Females	**4**
2 (1)	Epigastric region strongly elevated (Fig. 1F); bulb triangular (Fig. 1H, J)	***K.longling* sp. nov.**
—	Epigastric region flat; bulb rectangular (Tong et al. 2018: figs 2A, 4I)	**3**
3 (2)	Postgastric scutum with a cluster of strong, long setae; psembolus with collapsed lobe (Tong et al. 2018: figs 4E, 5B)	***K.mahmolae* Tong & Li, 2018**
—	Postgastric scutum without cluster of strong, long setae; psembolus with flat, wide and elongated lobe (Tong et al. 2018: figs 1C, 2B)	***K.putao* Tong & Li, 2018**
4 (1)	Postgastric scutum heart shaped (Fig. 3C)	***K.longling* sp. nov.**
—	Postgastric scutum rectangular (Tong et al. 2018: figs 3G, 6C)	**5**
5 (4)	Endogyne with crescent-shaped plate (Tong et al. 2018: fig. 3G)	***K.putao* Tong & Li, 2018**
—	Endogyne with triangular plate (Tong et al. 2018: fig. 6G)	***K.mahmolae* Tong & Li, 2018**

#### ﻿ Kachinia longling

Taxon classificationAnimaliaAraneaeOonopidae

Tong & Zhang sp. nov.

83FC57DD-B207-51D0-B931-DD9CFD1538A0

https://zoobank.org/651BE492-19F3-480A-9EB7-CF6675FCFF21

Figs 1 , 2 , 3 , 7

##### Type material.

***Holotype*** ♂ (SYNU-640), China, Yunnan Prov., Baoshan City, Longling Co., Longxin Town, Xiaoheishan Nature Reserve, 16.02.2011, Z. Li & L. Wang; ***Paratypes***: 1♀ (SYNU-641), 1♀ (SYNU-642), 4 ♀ (SYNU-643-646), same data as for the holotype.

##### Diagnosis.

**Figure 1.**
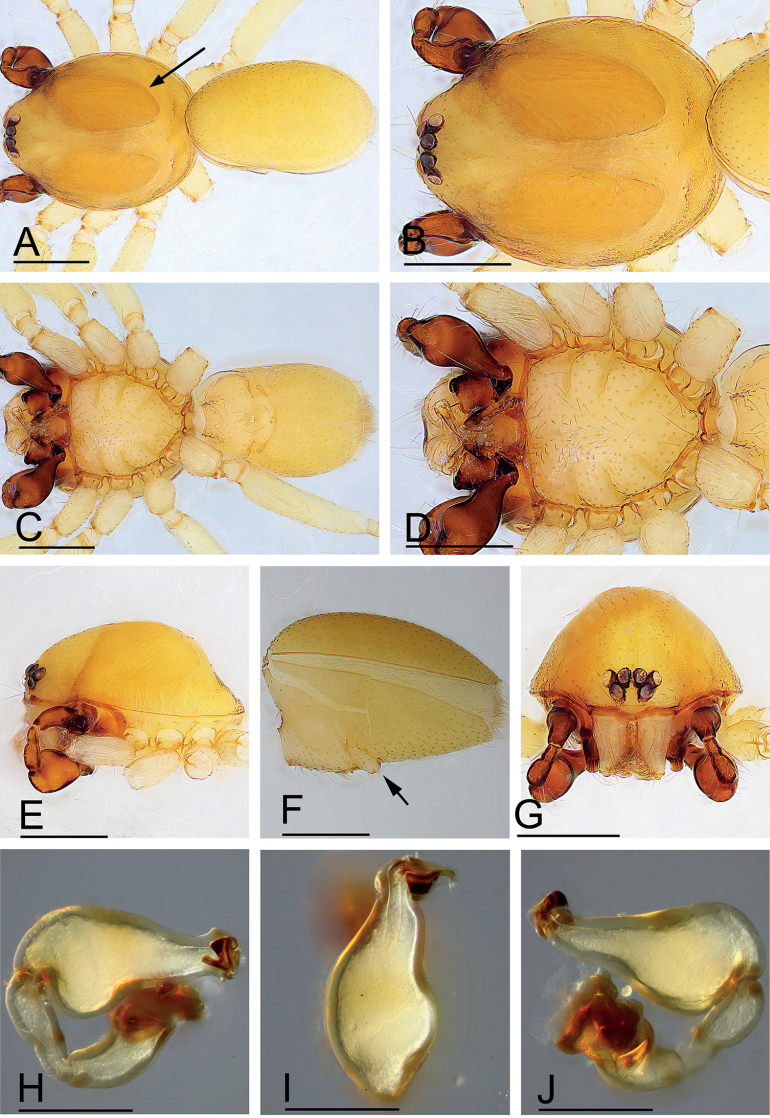
*Kachinialongling* sp. nov., holotype male **A, C** habitus (dorsal and ventral views), arrow shows the large coxal apodemes **B, D, E, G** prosoma (dorsal, ventral, lateral and anterior views) **F** abdomen, lateral view (arrow shows the strongly elevated epigastric region) **H–J** left palp (prolateral, dorsal and retrolateral views). Scale bars: 0.40 (**A–G**); 0.20 (**H–J**).

**Figure 2.**
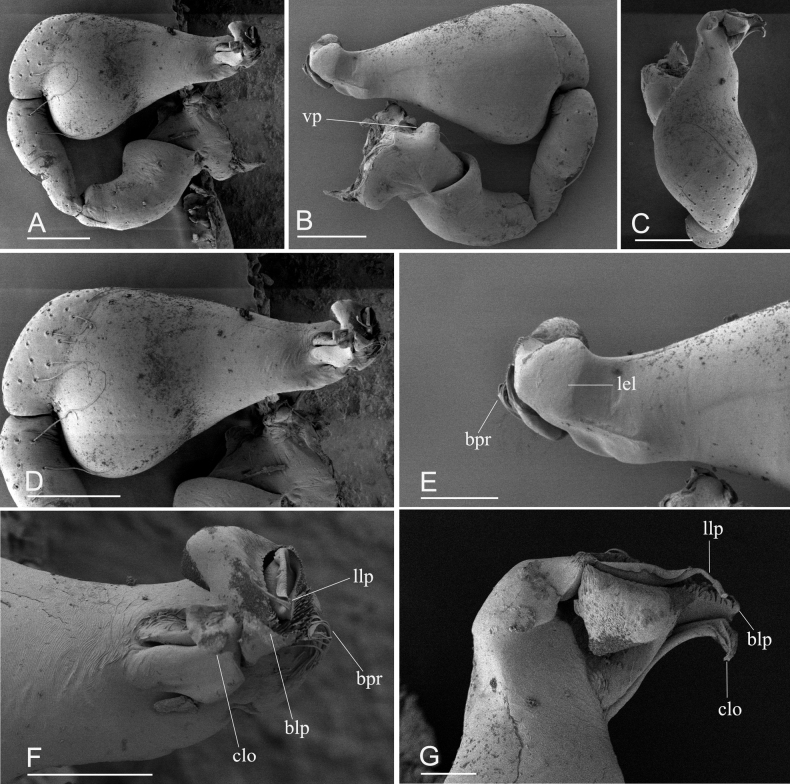
*Kachinialongling* sp. nov., holotype male, SEM**A, B** left palp (prolateral and retrolateral views) **C, D** palpal bulb (dorsal and prolateral views) **E–G** distal part of palpal bulb (retrolateral, prolateral and dorsal views). Abbreviations: blp = broom-like projection; bpr = brush-like projection; clo = curved lobe; lel = large, ear-shaped lobe; llp = leaf-like projection; vp = ventral protuberance. Scale bars: 0.10 (**A–D**); 0.05 (**E–G**).

**Figure 3.**
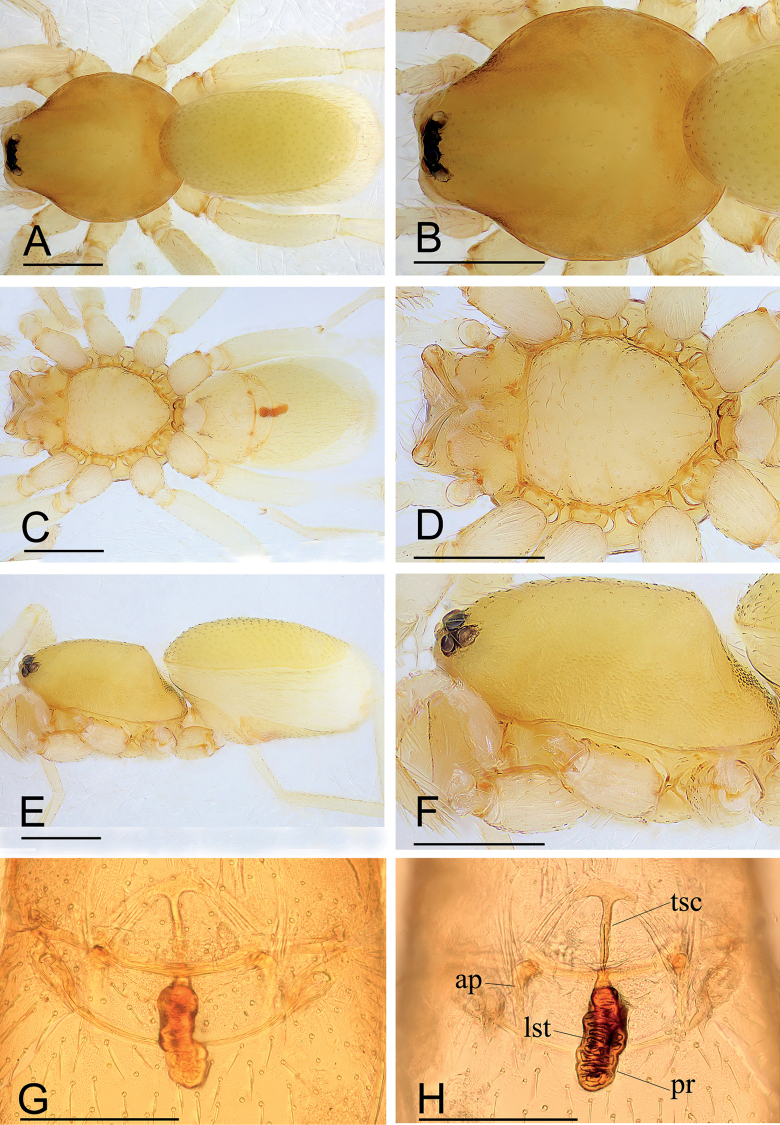
*Kachinialongling* sp. nov., paratype female **A, C, E** habitus (dorsal, ventral and lateral views) **B, D, F** prosoma (dorsal, ventral and lateral views) **G, H** copulatory organ (ventral and dorsal views). Abbreviations: ap = apodeme; lst = line-like structure; pr = posterior receptacle; tsc = T-shaped sclerite. Scale bars: 0.40 (**A–F**); 0.20 (**G–H**).

The new species can be distinguished from *K.mahmolae* and *K.putao* by the strongly elevated epigastric region of the male (arrow in Fig. 1F) vs. flat (see Tong et al. 2018: figs 1C, E, 4E, G), triangular bulb (Fig. 2A, B) vs. nearly rectangular (see Tong et al. 2018: figs 2A, 4I), and the nearly heart-shaped postgastric scutum of the female (Fig. 3C) vs. rectangular (see Tong et al. 2018: figs 3G, 6G).

**Figure 4.**
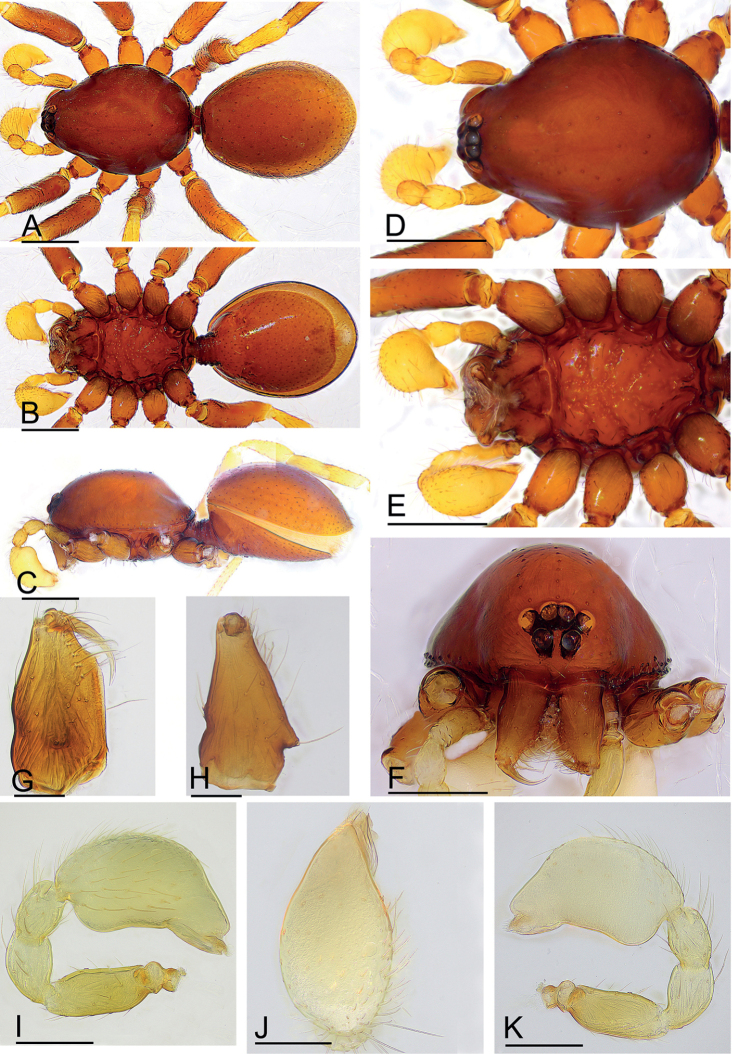
*Promolotralushui* sp. nov., holotype male **A–C** habitus (dorsal, ventral and lateral views) **D–F** prosoma (dorsal, ventral and anterior views) **G, H** left chelicera (anterior and lateral views) **I–K** left palp (prolateral, dorsal and retrolateral views). Scale bars: 0.40 (**A–F**); 0.20 (**I–K**); 0.10 (**G, H**).

##### Description.

**Male** (holotype). Habitus as in Fig. 1A, C. Body length 1.96; carapace 1.03 long, 0.86 wide; abdomen 1.01 long, 0.59 wide. Body yellow, legs lighter. Carapace (Fig. 1B, E): broadly oval in dorsal view, with large brown oval patches (coxal apodemes) behind eyes, longer than ½ of carapace; pars cephalica strongly elevated, pars thoracica higher than pars cephalica, surface of pars cephalica smooth; lateral margin straight, smooth, rebordered. Mouthparts (Fig. 1D, G): chelicerae straight; labium rectangular, anterior margin deeply incised; endites broad, distally branched. Abdomen (Fig. 1A, C, F): ovoid; booklung covers smooth; sperm pore small, oval, situated at level between anterior spiracles; anterior and posterior spiracles connected by furrow; epigastric region strongly elevated; dorsal scutum covering entire dorsum, strongly sclerotized; epigastric scutum strongly sclerotized, surrounding pedicel; postgastric scutum strongly sclerotized, long, almost rectangular, covering nearly the full length of the abdomen, with posteriorly-directed lateral apodemes. Palp (Figs 1H–J, 2A–G): strongly sclerotized; trochanter with a ventral protuberance (vp); femur 0.23 long, 0.14 width, length/maximal width = 1.64; cymbiobulb 0.41 long, 0.16 wide, length/maximal width = 2.56; psembolus complex (Fig. 2E, F, G) with flat, wide and elongated, strongly curved lobe (clo), leaf-like projection (llp), broom-like projection (blp), and long, brush-like projection (bpr) in prolateral view; with large ear-shaped lobe (lel) in retrolateral view.

**Female.** As in male except as noted. Habitus as in Fig. 3A, C, E. Body length 1.93; carapace 1.51 long, 0.78 wide; abdomen 1.15 long, 0.62 wide. Labium and endites unmodified. Abdomen (Fig. 3A, C): dorsal scutum large, covering more than 5/6 of dorsum; postgastric scutum heart shaped. Epigastric area (Fig. 3C, G): surface unmodified. Endogyne (Fig. 3H): with T-shaped sclerite (tsc) anteriorly, followed posteriorly by tube-like posterior receptacle (pr).

##### Etymology.

The specific name is a noun in apposition taken from the type locality.

##### Distribution.

Known only from the type locality.

#### ﻿Genus Promolotra

Taxon classificationAnimaliaAraneaeOonopidae

Tong & Li, 2020

956B765C-34F2-50EC-8981-3DD686DC17FA

##### Type species.

*Promolotrashankhaung* Tong & Li, 2020 from Myanmar.

##### Comment.

The genus belongs to the subfamily Oonopinae Simon, 1890. According to Tong and Li (2020), the genus is similar to *Molotra* Ubick & Griswold, 2011 by the heavily sclerotized dorsal and ventral abdominal scuta, the long spines on legs I and II, and the embolar region.

##### Composition.

*Promolotrahponkanrazi* Tong & Li, 2020, *P.lushui* sp. nov., *P.shankhaung* Tong & Li, 2020.

##### Distribution.

China (Yunnan), Myanmar.

### ﻿Key to *Promolotra* species (female of *P.hponkanrazi* unknown)

**Table:** 

1 (0)	Males	**2**
—	Females	**4**
2 (1)	Cymbiobulb apically with blunt end; dorsal lobe of psembolus with triangular extension (Fig. 5F)	***P.lushui* sp. nov.**
—	Without above-mentioned characters	**3**
3 (2)	Tibiae dark proximally; embolar region with narrow ventral lobe (Tong and Li 2020: figs 1D, 2E)	***P.shankhaung* Tong & Li, 2020**
—	Tibiae uniformly yellowish brown; embolar region with broad ventral lobe (Tong and Li 2020: figs 5E, 6E)	***P.hponkanrazi* Tong & Li, 2020**
4 (1)	Endogyne with posterior receptacle (Tong and Li 2020: fig. 4G)	***P.shankhaung* Tong & Li, 2020**
—	Endogyne without posterior receptacle (Fig. 6H)	***P.lushui* sp. nov.**

#### ﻿ Promolotra lushui

Taxon classificationAnimaliaAraneaeOonopidae

Tong & Zhang sp. nov.

0111FE05-4B7A-59C2-8DCA-D3F41A907301

https://zoobank.org/E02029CB-E8DA-471D-9B13-A5D39E3E20C0

Figs 4 , 5 , 6 , 7

##### Type material.

***Holotype*** ♂ (SYNU-647), China: Yunnan Prov., Lushui City, Pianma Town, 3.03.2011, Z. Li & L. Wang leg.; ***Paratypes***: 2 ♀ (SYNU-648-649), 3 ♂ (SYNU-650-652), same data as for the holotype.

##### Diagnosis.

**Figure 5.**
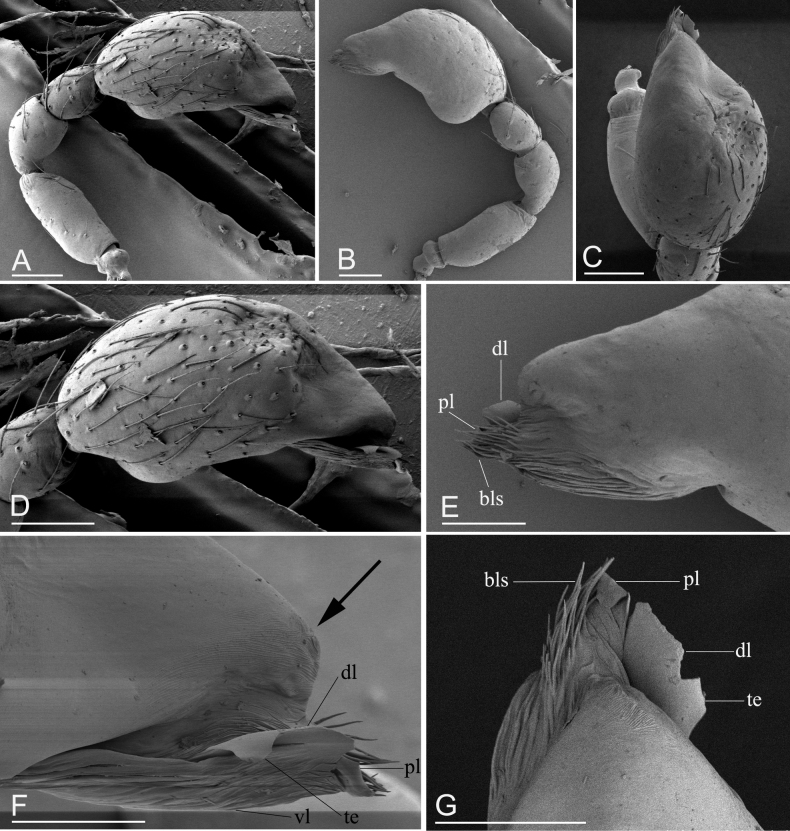
*Promolotralushui* sp. nov., holotype male, SEM**A–B** left palp (prolateral and retrolateral views) **C–D** palpal bulb (dorsal and prolateral views) **E–G** distal part of palpal bulb (retrolateral, prolateral and dorsal views). Abbreviations: bls = brush-like structures; dl = dorsal lobe; pl = posterior lobe; te = triangle extension; vl = ventral lobe. Scale bars: 0.10 (**A–D**); 0.05 (**E–G**).

**Figure 6.**
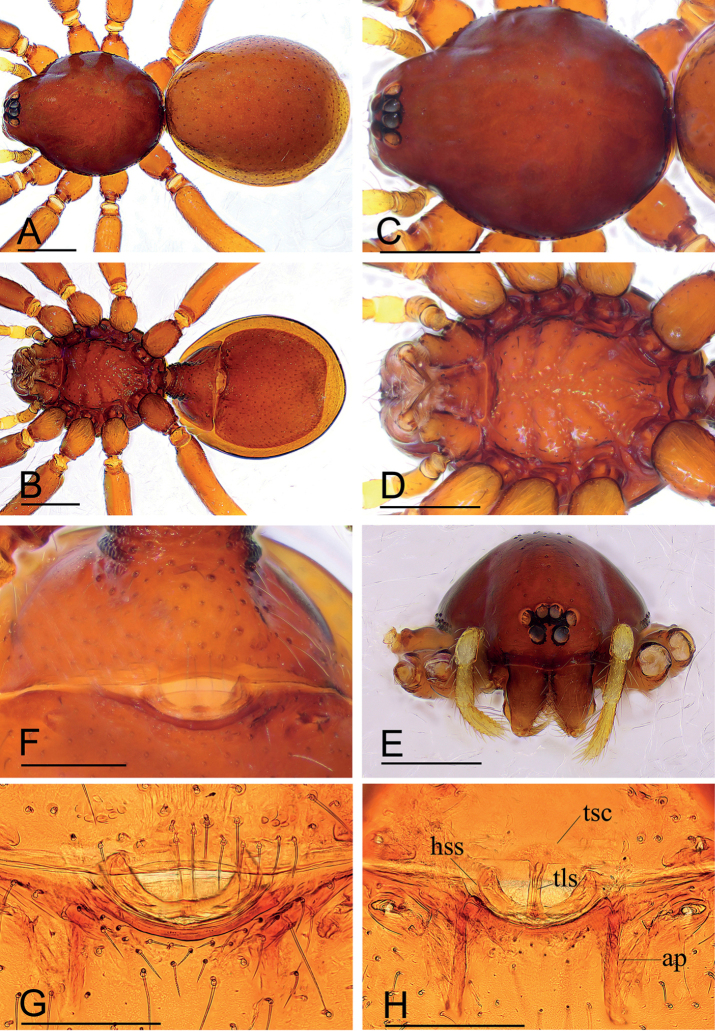
*Promolotralushui* sp. nov., paratype female **A, B** habitus (dorsal and ventral views) **C–E** prosoma (dorsal, ventral and anterior views) **F** copulatory organ, ventral view **G, H** copulatory organ (cleared in lactic acid, ventral and dorsal views). Abbreviations: ap = apodeme; hss = horseshoe-shaped sclerite; tls = tube-like structure; tsc = T-shaped sclerite. Scale bars: 0.40 (**A–E**); 0.10 (**G, H**).

The new species can be distinguished from congeners by the blunt end of the cymbiobulb (arrow in Fig. 5F) vs. lacking a blunt end (see Tong and Li 2020: figs 2A, 6A), triangular extension of the dorsal lobe of the embolar region (‘te’ in Fig. 5F) vs. lacking a triangular extension (see Tong and Li 2020: figs 2E, 6H), and absence of a posterior receptacle (Fig. 6H) vs. with a narrow posterior receptacle (see Tong and Li 2020: fig. 4G).

##### Description.

**Male** (holotype). Habitus as in Fig. 4A, B, C. Body length 2.31; carapace 1.09 long, 0.81 wide; abdomen 1.11 long, 0.87 wide. Body yellow-brown, legs yellow. Carapace (Fig. 4D, F): oval in dorsal view, without any pattern; pars cephalica slightly elevated in lateral view, surface of pars cephalica smooth; lateral margin straight, rebordered, with small blunt denticles. Eyes (Fig. 4D, F): ALE largest, PME, PLE subequal; ALE separated by nearly more than their radius, ALE–PLE separated by less than ALE radius, PME touching each other; posterior row recurved from above, procurved from front. Clypeus (Fig. 4F): ALE separated from edge of carapace by 2 diameters. Mouthparts (Fig. 4E–H): chelicerae straight, anterior face strongly swollen, with cone-shaped protuberance in lateral view; labium rectangular, anterior margin deeply incised; endites with distal excavation. Sternum (Fig. 4E): with radial furrows between coxae, surface smooth, covered with large, round pits; setae sparse, dark, needlelike, evenly scattered. Abdomen (Fig. 4A–C): booklung covers brown; sperm pore small, oval, rebordered, situated between anterior and posterior spiracles; dorsal scutum strongly sclerotized, covering whole abdomen; postgastric scutum strongly sclerotized, covering nearly full length of abdomen, with posteriorly-directed lateral apodemes. Palp (Figs 4I–K, 5A–G): pale orange; femur 0.26 long, patella 0.16 long, tibia 0.13 long, cymbiobulb 0.41 long, 0.22 wide, length/maximal width = 1.86; embolar region with flat dorsal lobe (dl), small posterior one (pl), and narrow, leaf-like, wrinkled ventral one (vl), with brush-like structures (bls) in retrolateral view.

**Female.** As in male except as noted. Habitus as in Fig. 6A, B. Body length 2.51; carapace 1.16 long, 0.92 wide; abdomen 1.38 long, 1.12 wide. Epigastric area (Fig. 6F, G): ‘atrium’ relatively wide, broadly oval. Endogyne (Fig. 6H): with a T-shaped sclerite (tsc) anteriorly, very thin, long and tube-like structure (tls) can be seen inside T-shaped sclerite; with horseshoe-shaped sclerite (hss) medially; apodemes (ap) well developed.

**Figure 7.**
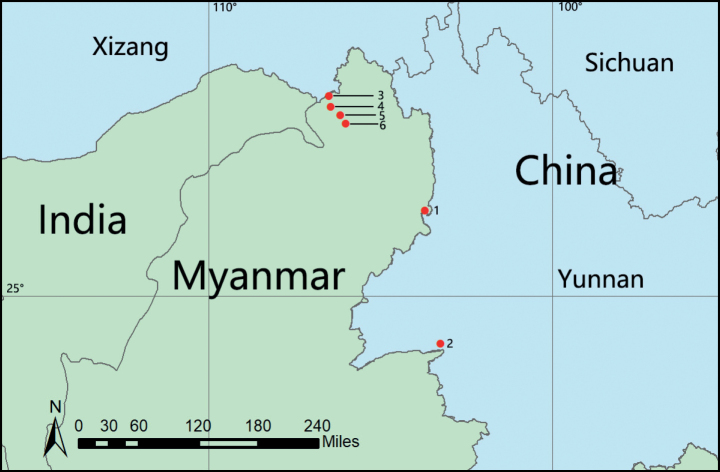
Distribution records of the species of *Kachinia* and *Promolotra* from Yunnan, China and Myanmar. 1. *K.longling* sp. nov.; 2. *P.lushui* sp. nov; 3. *P.hponkanrazi* Tong & Li, 2020; 4. *K.putao* Tong & Li, 2018; 5. *P.shankhaung* Tong & Li, 2020; 6. *K.mahmolae* Tong & Li, 2018.

##### Etymology.

The specific name is a noun in apposition taken from the type locality.

##### Distribution.

Known only from the type locality.

## Supplementary Material

XML Treatment for Kachinia

XML Treatment for Kachinia longling

XML Treatment for Promolotra

XML Treatment for Promolotra lushui

## ﻿Additional information

### Author ORCIDs

Shuhui Li https://orcid.org/0009-0000-0266-6859

Dongju Bian https://orcid.org/0000-0002-9324-8026

Yanfeng Tong https://orcid.org/0000-0002-4348-7029

Zhisheng Zhang https://orcid.org/0000-0002-9304-1789

### Data availability

All of the data that support the findings of this study are available in the main text.
